# Bile Acids in Dementia: Brain Amyloid, White Matter Lesions, and Atrophy

**DOI:** 10.1093/geroni/igaa057.2772

**Published:** 2020-12-16

**Authors:** Vijay Varma, Youjin Wang, Yang An, Sudhir Varma, Murat Bilgel, Jimit Doshi, Cristina Legido-Quigley, Madhav Thambisetty

**Affiliations:** 1 National Institute on Aging, Bethesda, Maryland, United States; 2 NCI, Bethesda, Maryland, United States; 3 HiThru Analytics, Laurel, Maryland, United States; 4 UPenn, Philadelphia, Pennsylvania, United States; 5 King’s College London, London, England, United Kingdom; 6 NIA, Baltimore, Maryland, United States

## Abstract

While Alzheimer’s disease (AD) and vascular dementia (VaD) may be accelerated by hypercholesterolemia, the mechanisms underlying this association is unclear. Using a novel, 3-step study design we examined the role of cholesterol catabolism in dementia by testing whether 1) the synthesis of the primary cholesterol breakdown products (bile acids (BA)) were associated with neuroimaging markers of dementia; 2) pharmacological modulation of BAs alters dementia risk; and 3) brain BA concentrations and gene expression were associated with AD. We found that higher serum concentrations of BAs are associated with lower brain amyloid deposition, slower WML accumulation, and slower brain atrophy in males. Opposite effects were observed in females. Modulation of BA levels alters risk of incident VaD in males. Altered brain BA signaling at the metabolite and gene expression levels occurs in AD. Dysregulation of peripheral cholesterol catabolism and BA synthesis may impact dementia pathogenesis through signaling pathways in the brain.

